# Asylums of the World


**Published:** 1892-07-02

**Authors:** 


### INSTITUTION LITERATURE.

### ASYLUMS OF THE WORLD.*

### I.—HISTORY AND EARLY TREATMENT.

THE subject of insanity and its treatment, has in all ages attracted much attention. With the record of the past behind us, we can to-day review, as from a high point of observation, the forms of insanity from early times, the failures and development of treatment, the superstitions and atrocities connected with it, and thus form a judgment in the light of the past and the present. The subject is one of ever-growing interest alike to the specialist and the lay public. Mr. Burdett in his large and interesting work on the insane, their treatment and habitation, has given us an opportunity of thoroughly covering the whole field. The volumes, if regarded as books of reference alone, should be included in all institution libraries. For the general reader we give a sketch of many of the points of interest connected with the insane in all lands to be found in the work.

The author acknowledges the assistance of Dr. Green, of the Northampton Asylum, and of Dr. Sibbald, Commissioner in Lunacy for Scotland, and in his preface recounts some of the difficulties which he had to overcome in collecting the necessary materials for his undertaking. The first three or four chapters of the first volume give a most interesting and instructive account of insanity in pre-Christian times. Beginning with the mythical Greek and Roman, and passing through the Assyrian, Egyptian, and Hebrew periods, the history of the treatment of the insane is gradually unfolded down to the middle of the present century. The very earliest case in which medicine was administered to a patient suffering from insanity is recorded, and the drugs prescribed included hellebore, and were given by the Greek physician, Melampus, to the three daughters of Proetus, King of Argolis. We have further information by means of the erudite researches of Dr. Sibbald into the dramatic writings of early Greece, especially in the works of Euripides. This writer describes the mental derangement of his hero, Orestes, which he pictures as passing from remorse into deep and morbid depression, ending in an explosion of maniacal excitement. Aristophanes also describes a case of monomania. The patient was locked up, bathed and purged, with no avail. These clinical descriptions leave no doubt that madness was recognised by the Greeks as a true physical disease, removable by therapeutical and other agencies, and for the relief of which the best medical skill was retained.

Proceeding from the enlightened treatment of Greece to the still more civilised condition of the Roman Republic, we find not only an intelligent and advanced medical view of the nature of insanity, but also legislative enactments not at the present time superseded in some respects by the laws of the leading nations of modern Europe. We select the following: "Mad men (*furiosi*) and lunatics (*menti capti*) were not answerable for any wrongful act which they committed because they were deemed to be incapable of having a wrong intention."

"Madmen always had curators, even if they were more than twenty-five years of age."

"A person sometimes insane could when in a rational mood bind himself and others by contract." We are told that the closest and most diligent research has failed to bring to light any evidence of the confinement of insane patients, or of their treatment together in assigned places; but it is probable that the temples were used largely for the treatment of acute cases of insanity, and that the more violent and dangerous cases were confined in the jails and prisons.

A few stray phrases gathered from the Roman writers

* "Hospitals and Asylums of the World." By Henry C. Burdett. Vols. I. and II.—Asylums and Asylum Construction. (London: J. and A. Churchill).

is sufficient to throw considerable light upon the medical treatment of the insane under the Romans. There is no doubt that diet and bathing, with the administration of hellebore, constituted a considerable portion of the early treatment, and instances might be quoted of patients sent to Epidaurus for treatment in the temple of Æsculapius. This temple had a hydropathic establishment attached to it, and those troubled in mind were frequently sent to Epidaurus to undergo a course of treatment . . . . Dr. Sibbald mentions a house situated in the Piraeus which was said to be used for the same purpose."

The ancient Assyrian records have failed to reveal any allusion to the treatment of disease and of insanity in particular. Every temple appears to have had its laboratories where the remedies were prepared. Pinel, in his treatise on insanity, gives the following glowing description of the treatment of the insane by the priests of the temple of Saturn in Egypt: "Games and recreations of all kinds were instituted in these temples. Flowery gardens and groves disposed with taste and art invited them to refreshment and salubrious exercise. Gaily decorated boats sometimes transported them to breathe amidst rural concerts the pure breezes of the Nile. Every moment was devoted to some pleasurable occupation, or rather, a system of diverting amusements enhanced and sanctioned by superstition. An appropriate and scrupulously observed regimen, repeated excursions, with every advantage of a similar nature that the experienced priesthood could invent or command, were in no small degree calculated to suspend the influence of pain, to calm the inquietude of a morbid mind, and to operate salutary changes in the various functions of the system."

The early Christian epoch continues to furnish us with the same enlightened views of the origin and nature and treatment of insanity, as we have quoted from the writings of the Greeks and early Romans. Particularly are we struck with the singularly advanced views of Soranus, who flourished a few years before the reign of Trajan, 95 or 96 A.D. He appears to have freed himself from the mass of superstition which in every age is apt to grow around the subject of insanity. He discriminated the various forms of delusions to which his patients were subject, and was able to diagnose and classify most of the forms of insanity. Above all, his treatment of the insane was characterised by that humanity, carefulness, and discretion which, we regret to say, is not yet the rule in every Christian country. The first principle of Soranus' treatment seems to have been to remove from his patient every possible cause of irritation, external and internal. In acute cases according to the course of the disease, both the dietary and the various forms of remedial applications and therapeutic measures were carefully and gradually increased or decreased. Some of these measures, though entirely empirical, appear to us, with our present knowledge, to have a sound, scientific basis. For instance, the application of scarified cuppings to the lower part of the neck and shoulders as a relief to cerebral congestion, and the application of warm sponges to the eyelids after long deprivation from sleep as a soporific medium, are extremely interesting to the modern physiologist and physician.

A certain amount of enlightened care for the insane appears to have characterised the early civil Christian nations from the first down to the seventh century of our era, and we are led to believe that certain specially equipped hospitals for the treatment of lunatics existed in noted centres, for instance at Jerusalem and Constantinople.

After the year 600 we enter, not without a shudder, upon a period of 1,250 years stigmatised by the heartless cruelty of humanity towards those afflicted members of the race. It is true that the atrocious barbarities exercised upon the insane are attributable chiefly to the grossest superstition openly sanctioned by the Church—to the thoughtless ignorance of the people at large, and to the animal instinct which seems to take pleasure in the maltreatment by the stronger of the weaker or deformed members of a species. But when we consider that it is only fifty years since a more humane method of treatment began to raise itself out of the ashes of the old system, and further that it is only under the compulsion of stringent penal enactments that the ill-treatment of the insane is being at present repressed in the most civilised countries of Europe, we are compelled to admit that, in this respect at least, our boasted civilisation has only begun to dawn.

The period of demoniacal possession extended from the year 600 to the middle of the eighteenth century. It is true, as we are told in Chapter II. of this work, that the condition of the people throughout all Europe was more miserable than at any other period of history. In the year 630 A.D., the physician Paulus Aegineta raised a final protest against the miraculous and supernatural opinions which were being entertained towards insanity. His voice was drowned in religious superstition, and it was not for nearly 1,200 years thereafter that the great French physician, Pinel, boldly proclaimed to the world the wisdom of the principles originally laid down by Paulus Aegineta.

A quotation from a French author throws light upon the attitude of the learned and unlearned among the people towards lunatics. "We may say that doctors shared the insanity of the maniacs. The lunatic was no longer a patient, he was no longer even a man, but a kind of wild and formidable beast, half animal, half demon. In the horror that he inspired they declared him to be possessed of Satan, and threw him into the flames. Since during these times the State made no provision for the support of the maniac, his relatives, no doubt with an eye to the main chance, readily acquiesed in the justice of the sentence of mother church, which condemned the lunatic to the stake."

The period which the author calls that of "brutal suppression" and ill-treatment began with the growth of the establishments for the care of the insane. The horrors of the system of suppression as a means of treatment were thrown into strong relief by the humane actions of Pinel in France and Luke and Conolly in England; further, a lurid glare is thrown upon the system by the investigations of the Royal Commissions of England in 1846 and in Scotland in 1855. At page 66 we are told "the general employment of chains was revolting. The patients had collars and belts of iron and fetters on their hands and feet. Some were fastened to the wall by a chain a foot and a half long, and this method was extolled as being peculiarly calming. The patients were usually without fresh air, without light, without water to allay their thirst—under the dominion of gaolers who were frequently criminals of the worse type, and chained in caves which would not have been thought good enough for wild beasts. In a large private asylum near London several of the passive women were chained to their bedsteads naked, or only covered with a hempen rug, and this in the month of December. One towel a week was accorded for the use of 170 patients, and some were mopped with cold water in the severest weather. The effect of this treatment upon the administrators of asylums is said by Conolly to have been most debasing and brutalising. Some of the doctors took a fiendish pleasure in torturing their patients, under the name of medical treatment, by administering cold douches, surprise baths, and tying them in rapidly rotating chairs until the victims became insensible. Many of the keepers were brutalised villains of the lowest description, and the female keepers slovenly, unsexed, and debased.

